# Markedly elevated blood pressure, tachycardia, and altered consciousness in patients with bacteremia during transurethral surgeries: two case reports

**DOI:** 10.1186/s40981-025-00774-z

**Published:** 2025-02-17

**Authors:** Arisa Hotta, Momoka Nishimura, Daisuke Nakada, Riko Uchida, Hiroshi Matsuura, Naoko Torii, Naoko Fujita, Taku Hamada, Ai Nakamoto, Noriko Yoshikawa

**Affiliations:** Department of Anesthesiology, Sumitomo Hospital, 5-3-20 Nakanoshima, Kita-Ku, Osaka City, Osaka, 530-0005 Japan

**Keywords:** Bacteremia, Elevated blood pressure, Altered consciousness, Transurethral surgery

## Abstract

**Background:**

Transurethral surgery is often accompanied by postoperative urinary tract infection. Although early detection and adequate treatment of bacteremia are required to prevent sepsis, it is usually undetectable during surgery. We report two cases with remarkable hypertension and tachycardia during transurethral surgery in which bacteremia was diagnosed by an intraoperative blood test.

**Case presentation:**

An 80-year-old man (Case 1) underwent transurethral holmium laser prostate enucleation under spinal anesthesia, and an 88-year-old woman (Case 2) underwent transurethral resection of bladder tumor under general anesthesia. Altered consciousness (Case 1) and postoperative delirium (Case 2) were noted, in addition to remarkable intraoperative hypertension and tachycardia. We administered broad-spectrum antibiotics for possible bacteremia in both cases. The patients’ hemodynamics positively recovered the following day. Intraoperative blood samples revealed gram-negative bacillus.

**Conclusions:**

Hypertension, tachycardia, and altered consciousness may suggest the onset of symptomatic bacteremia during transurethral surgery, and adequate treatment is required to prevent sepsis.

## Background

Urinary tract infection is a representative complication following transurethral surgery. Despite a high incidence of perioperative onset of bacteremia [1], which may result in sepsis and lead to poor prognosis, patients are usually asymptomatic during surgery. We report two cases of symptomatic bacteremia with altered consciousness and remarkable increases in blood pressure (BP) and heart rate (HR) during or immediately after transurethral surgery, which required hemodynamic control and antibiotic therapy.

## Case presentation

### Case 1

**Fig. 1:**
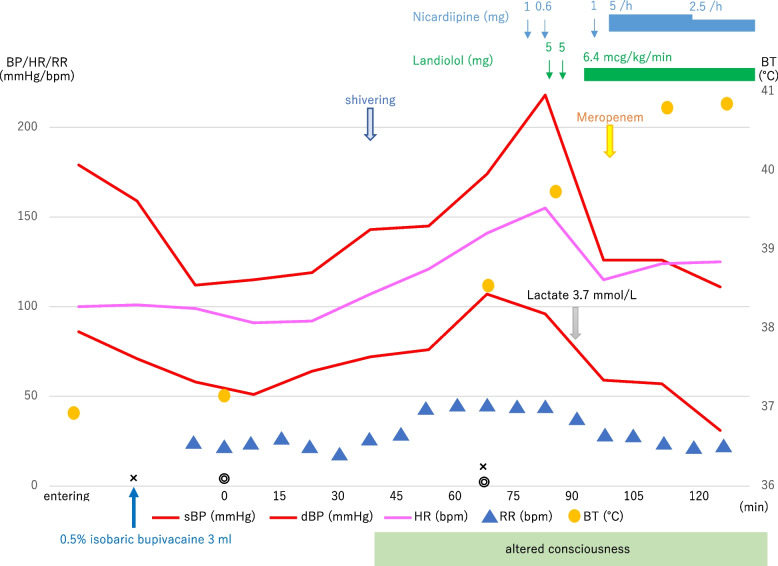
Hemodynamics during transurethral morcellation in Case 1. In the middle of surgery under spinal anesthesia, the patient complained of chills with severe shivering and tachycardia, which exacerbated after surgery. Abbreviations: × , anesthesia start/end; concentric circle, operation start/end; sBP, systolic blood pressure; dBP, diastolic blood pressure; HR, heart rate; RR, respiratory rate; BT, body temperature

An 80-year-old man was scheduled to undergo holmium laser enucleation of the prostate for benign prostatic hypertrophy. He had undergone repeated urinary catheterizations for 4 months for urinary retention without urine cultures. Despite never having been diagnosed with hypertension, his BP on admission was approximately 160/85 mmHg. Laboratory tests showed leukocytes of 7400/μL and C-reactive protein (CRP) of 0.17 mg/dL. An electrocardiogram and echocardiography were unremarkable. A two-stage procedure was required due to significant prostate volume. The first surgery was performed under general anesthesia and induced with propofol, remifentanil, and rocuronium and maintained with desflurane, remifentanil, rocuronium, and fentanyl. Intraoperative BP, HR, and body temperature (BT) were 85–173/47–84 mmHg, 50–60 bpm, and approximately 37.0 °C, respectively. After surgery, the enucleated prostate tissue was retained, and a urinary catheter was left for continuous bladder lavage. On the next day, laboratory data showed an increase in leukocytes (15,700/μL) and CRP (3.84 mg/dL), considered as the effect of surgery. Cefazolin was discontinued, and the peripheral venous catheter was removed as planned. Urine cultures and another blood test were not performed until the second surgery because of no fever. After 8 days, transurethral morcellation was performed under spinal anesthesia. Upon entering the operating room, BP of 179/86 mmHg, HR of 100 bpm, BT of 36 °C, and respiratory rate (RR) of 18 bpm were noted. Spinal anesthesia with 3 ml of 0.5% isobaric bupivacaine from the L 3/4 interspace provided analgesia below the Th 11 level bilaterally (Fig. 1). At 38 min after the start of surgery, the patient complained of chills with severe shivering. Hypoalgesia level was bilaterally up to Th 6. He was unable to communicate adequately due to shivering and agitation. The surgery was terminated at 71 min. The patient’s BP, HR, BT, and RR continued to rise, peaking at 218/96 mmHg, 153 bpm, 40.9 °C, and 41 bpm, respectively. His lactate level was 3.7 mmol/L. We performed blood cultures and various hormonal tests with suspicion of bacteremia and hormonal abnormalities and administered 1 g meropenem. Nicardipine and landiolol administration were required.

Immediately after admission to the intensive care unit (ICU), BP of 108/78 mmHg, HR of 120 bpm, and BT of 40.5 °C were noted with 2.5 μg/kg/min landiolol. Altered consciousness improved. No antipyretic analgesics were administered during the ICU stay. On postoperative day (POD) 1, although laboratory data showed a remarkable increase in leukocytes (22,500/μL) and CRP (10.79 mg/dL), hemodynamics were stable with BP of 100/56 mmHg, HR of 66 bpm, and BT of 36.7 °C without any cardiovascular medications and landiolol. He was discharged from the ICU. The intraoperative blood culture revealed Acinetobacter baumannii, which was sensitive to meropenem. Intraoperative thyroid hormone levels were normal; thyroid-stimulating hormone (the International Federation for Clinical Chemistry and Laboratory Medicine) was 1.55 μIU/mL, free-triiodothyronine was 2.3 pg/mL, and free-thyroxine was 0.9 ng/dL. Intraoperative epinephrine and norepinephrine levels increased to 111 pg/mL and 840 pg/mL, respectively, and normalized to 26 pg/mL and 293 pg/mL on POD 1.

### Case 2

**Fig. 2:**
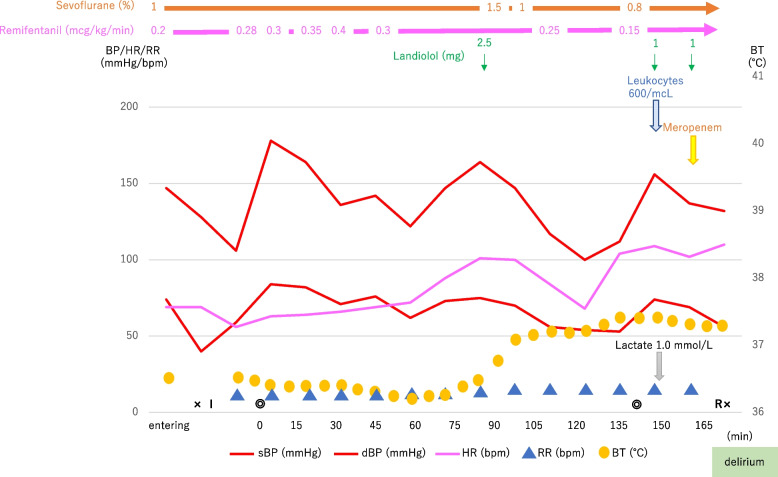
Hemodynamics during transurethral resection of bladder tumor in Case 2. Large dose of remifentanil was required to control hypertension during surgery under general anesthesia. Severe leukopenia was noted with hemodynamic changes at the end of surgery. Abbreviations: × , anesthesia start/end; concentric circle, operation start/end; sBP, systolic blood pressure; dBP, diastolic blood pressure; HR, heart rate; RR, respiratory rate; BT, body temperature; I, insertion of ProSeal® laryngeal mask airway; R, removal of ProSeal® laryngeal mask airway

An 88-year-old woman with bladder cancer was scheduled to undergo transurethral resection of a bladder tumor (TUR-BT). Her medical history included controlled hypertension with amlodipine besylate and untreated dyslipidemia. Chronic residual urine and pyuria were noted, but there was no fever. Preoperative laboratory data showed leukocytes of 6300/μL and CRP of 0.06 mg/dL. Because the tumor was large and its removal was expected to take a long time, the surgery was performed under general anesthesia. Upon entering the operating room, BP of 147/74 mmHg, HR of 69 bpm, and BT of 36.5 °C were noted. General anesthesia was induced with 6 L/min oxygen, 1 mg/kg propofol, 0.5 mg/kg rocuronium, and 0.2 μg/kg/min remifentanil, and a ProSeal® laryngeal mask airway (P-LMA) was inserted (Fig. 2). Anesthesia was maintained with 0.5 L/min oxygen, 2.5 L/min air, 0.8–1.5% sevoflurane, and 0.05–0.4 μg/kg/min remifentanil, and 10 mg rocuronium was added appropriately. BP was 178/84 mmHg at the beginning of surgery, and remifentanil 0.3–0.4 μg/kg/min was required for controlling BP. At 90 min, HR, BP, and BT gradually increased to 101 bpm, 163/77 mmHg, and 37.4 °C, respectively, and landiolol bolus 1–2.5 mg was required. Blood test at the end of surgery showed a remarkable decrease in leukocytes of 600/μL, and lactate level was 1.0 mmol/L. We performed blood cultures and administered 1 g meropenem. After the completion of the surgery, the patient recovered from general anesthesia, and the p-LMA was removed. She was unable to remain in a resting state due to her delirium.

On admission to the ICU, BP of 99/64 mmHg, HR of 106 bpm, BT of 38.5 °C, lactate level of 2.2 mmol/L, and disorientation were noted. Dexmedetomidine was administered at 0.1 μg/kg/h for suspected delirium. No antipyretic analgesics were administered during the ICU stay. On POD 1, laboratory data showed increases in leukocytes (12,100/μL) and CRP (6.57 mg/dL). Hemodynamics were stable without any cardiovascular medications: BP of 118/68 mmHg and HR of 89 bpm. However, physical cooling was applied because BT was 39.2 °C. Dexmedetomidine was discontinued after the improvement of delirium. The intraoperative blood culture revealed Escherichia coli. No other cultures were taken postoperatively.

## Discussion

We experienced two cases of bacteremia in which an increase in BP, tachycardia, and altered consciousness occurred during and after transurethral surgery. Neither patient had a venous catheter causing bloodstream infection before surgery. Preoperative bacteriuria and an indwelling urinary catheter are significantly related to the development of intraoperative bacteremia, although most cases are asymptomatic [1], suggesting that undiagnosed bacteremia was present preoperatively in our cases. In Case 1, intraoperative blood culture revealed Acinetobacter baumannii, an opportunistic pathogen among hospital patients [2]. Although the patient was not immunosuppressed, Acinetobacter from a urinary catheter or prostate tissue may have been growing preoperatively. Morcellation, which requires the bladder to be fully distended before the suction of tissue, might increase the risk of bacteremia [3]. Because of minimal invasion, short duration of surgery and urinary catheter placement, there are few reports on bacteremia during TUR-BT [4]. However, chronic urinary retention and resection of a large area of the bladder may have increased the risk of dissemination of bacteremia in Case 2. Although hemodynamic condition improved on the next day, preoperative antibiotics, considering urine cultures, could have prevented bacteremia.

We had difficulty in managing markedly elevated BP. In Case 1, we initially postulated hormonal abnormalities, including hyperthyroidism, but normal postoperative hormone levels ruled this out. Animal studies showed an increase in BP with increased plasma catecholamine levels and tachycardia shortly after bacterial invasion into the blood [5]. In contrast to sepsis, for which hypotension is a typical symptom [6], bacteremia accompanied by an increase in BP is rarely reported [7, 8]. Additionally, other animal studies suggested that severe leukopenia occurred within 3 h after the onset of bacteremia by a gram-negative bacillus [9, 10], as in Case 2, implying that bloodstream infection from bacteremia had just occurred at that moment.

Altered consciousness in both patients might be related to sepsis-associated delirium. Animal studies have shown that vascular endothelial damage in the brain after bacteremia may occur before or in the very early stages of sepsis [11, 12]. Elderly patients like our patients are particularly susceptible to sepsis-associated delirium [13]. In both patients, the restless state quickly recovered with improvement in their general condition.

There are limitations in our report. First, the patients underwent different types of anesthesia. General anesthesia makes it difficult to determine whether altered consciousness is from delirium, aging, or the effects of anesthesia. Furthermore, anesthetics affect hemodynamics, and BP in Case 2 was therefore lower than that in Case 1. Second, we did not perform the same kind of blood tests in each case, which might have provided more useful information. Third, there has been no study on hemodynamics and catecholamine changes in humans during deteriorating bacteremia. Responses to bacteremia may differ in humans and animals [14].

We were able to prevent the development of sepsis in two elderly patients by administering antibiotics at the moment when symptomatic bacteremia occurred. Both patients had elevated BP, tachycardia, and altered consciousness. Urine cultures and preoperative antibiotic therapy should be performed if necessary for patients who are scheduled for transurethral surgery.

## Data Availability

Not applicable.

## Abbreviations

BP: Blood pressure
HR: Heart rate
CRP: C-reactive protein
BT: Body temperature
RR: Respiratory rate
ICU: Intensive care unit
POD: Postoperative day
TUR-BT: Transurethral resection of bladder tumor
p-LMA: ProSeal® laryngeal mask airway
